# Facial Recognition in a Discus Fish (Cichlidae): Experimental Approach Using Digital Models

**DOI:** 10.1371/journal.pone.0154543

**Published:** 2016-05-18

**Authors:** Shun Satoh, Hirokazu Tanaka, Masanori Kohda

**Affiliations:** Laboratory of animal Sociology, Department of Biology and Geosciences, Osaka City University, Sugimoto, Sumiyoshi, Osaka city, Osaka, Japan; University of Sussex, UNITED KINGDOM

## Abstract

A number of mammals and birds are known to be capable of visually discriminating between familiar and unfamiliar individuals, depending on facial patterns in some species. Many fish also visually recognize other conspecifics individually, and previous studies report that facial color patterns can be an initial signal for individual recognition. For example, a cichlid fish and a damselfish will use individual-specific color patterns that develop only in the facial area. However, it remains to be determined whether the facial area is an especially favorable site for visual signals in fish, and if so why? The monogamous discus fish, *Symphysopdon aequifasciatus* (Cichlidae), is capable of visually distinguishing its pair-partner from other conspecifics. Discus fish have individual-specific coloration patterns on entire body including the facial area, frontal head, trunk and vertical fins. If the facial area is an inherently important site for the visual cues, this species will use facial patterns for individual recognition, but otherwise they will use patterns on other body parts as well. We used modified digital models to examine whether discus fish use only facial coloration for individual recognition. Digital models of four different combinations of familiar and unfamiliar fish faces and bodies were displayed in frontal and lateral views. Focal fish frequently performed partner-specific displays towards partner-face models, and did aggressive displays towards models of non-partner’s faces. We conclude that to identify individuals this fish does not depend on frontal color patterns but does on lateral facial color patterns, although they have unique color patterns on the other parts of body. We discuss the significance of facial coloration for individual recognition in fish compared with birds and mammals.

## Introduction

Animals that repeatedly encounter each other in communities structured by dominance hierarchies or territoriality often develop social signals facilitating the recognition of individuals [1–4]. Such signals may be beneficial for both signal senders and receivers on average [2]; for example, they may help to avoid costly physical fighting associated with social conflict. The rapidity and accuracy of recognition are critically important in such social interactions, and visual cues can be one of effective types of sensory signal in many types of social encounters [2]. Thus, visual signals for individual identification are common among mammals and birds, and a number of species use facial patterns as visual recognition cues (e.g. primates [5–7], sheep [8,9] and birds [10–12]).

Like social birds and mammals, many species of fish are capable of individual recognition [1,3,4,13–15]. Fish may have visual acuity comparable to humans [16], and it is reported that social fish can recognize individuals visually [3,14,15,17], although they may also use different types of sensory cues such as acoustic and olfactory [4]. Recently, it was demonstrated that individual variation in color patterns in facial area (around eye to cheek area on operculum) is used for individual recognition in the cooperative breeding cichlid, *Neolamprologus pulcher*: fish can quickly and accurately distinguish between familiar and unfamiliar fish based on small differences in facial coloration [15]. Comparative examination suggests other cooperative breeding cichlids may also develop individual-specific facial coloration, perhaps because quick individual discrimination will be advantageous [15]. Similarly, permanently territorial damselfish may recognize neighbor individuals based on their facial coloration, which varies among individuals [14,15,18].

Although the aforementioned fish have individual-specific facial coloration patterns, it is not clear whether the face should be an especially favorable site for visual cues, and if so why it is face. We proposed three hypotheses to explain why individual color signals of fish tend to develop in the facial area [15]: (1) ‘Encountering’ hypothesis: When fish encounter each other, they will often approach in a ‘head-to-head’ position, where signals on the frontal parts of the fish will be most effective. (2) ‘Main body’ hypothesis: The critical social signal should be on the main parts of the body, not on peripheral parts that could easily be damaged or lost in conflict, such as the fins. (3) ‘Face-specific’ (gazing-eye) hypothesis: The face will inevitably be the site of visual signals for individual recognition. If fish initially attend to the eyes of other individual fish, as documented in some primates, social signals near the eyes will facilitate rapid and accurate signaling for both senders and receivers. However, these hypotheses have not been tested well, partly because studies have examined fish species of which color signals are located only in the facial area [15]. We can test these hypotheses more thoroughly using fish that have signal coloration not only in their facial area, but also on other body parts: If facial coloration is the most important type, such a fish will also exclusively use facial patterns for individual recognition.

**Fig 1:**
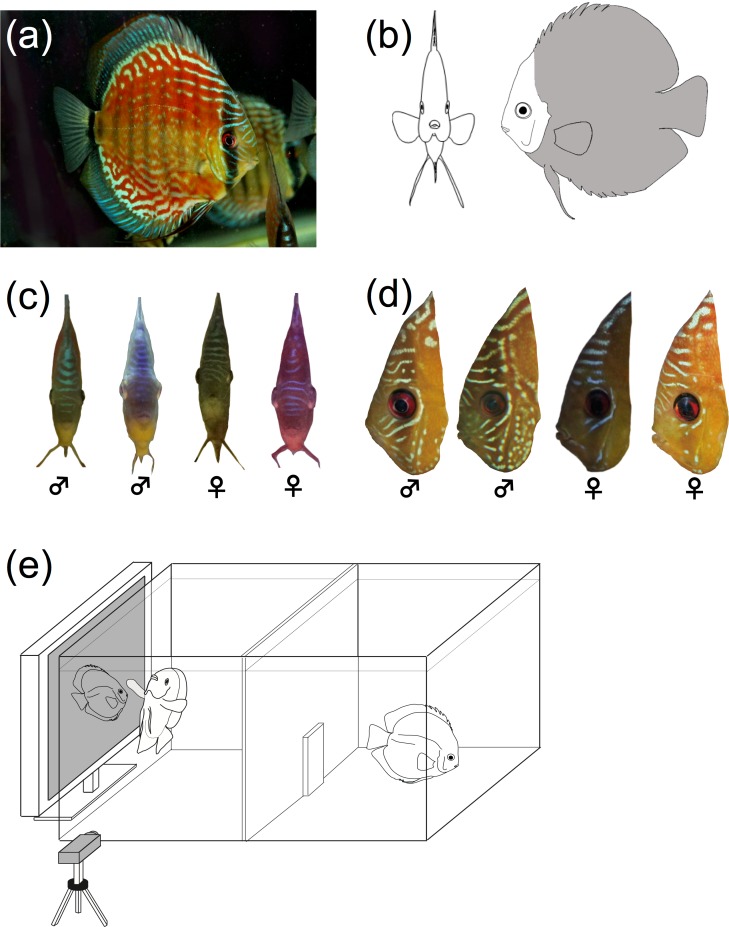
Coloration of the discus fish *Symphysopdon aequifasciatus* and experimental tank design. (a) Example of coloration on a whole fish body. (b) Facial area (white area) and other parts (gray area). (c) Examples of color variation on the head in frontal view and (d) facial color variation in lateral view. (e) Sketch of the experimental tank with video camera.

The discus fish *Symphysopdon aequifaciatus*, which inhabits South American waters, develops individual-specific color patterns on the facial area, front of the head, body trunk and vertical fins in both sexes [19] (Fig 1A). This bright and conspicuous coloration appears during the breeding phase after mating, and thus is unlikely to be influenced by sexual selection [20,21]. Individual coloration patterns persist for at least two years in comparison with the body colorations in photographs (Satoh, pers. obs.). The discus fish is monogamous, and is friendly to the partners but is aggressive against approaching non-partner fish during the breeding, indicating that they discriminates breeding partners from other conspecifics probably visually (Satoh, pers. obs.). Using the experimental methodology of Kohda et al. (2015) and the discus fish *S*. *aequifaciatus* from a wild population [22,23], we tested which coloration patterns these fish use for individual recognition. Here, we discuss our results as they pertain to the three facial coloration hypotheses and the evolution of facial coloration in fish.

## Materials and Methods

### Preparation of animal subjects

We obtained the discus fish *S*. *aequifasciatus* from two commercial shops, Pet Ballon, Osaka (http://www.petballoon.co.jp) and B-House, Hiroshima (https://www.youtube.com/watch?v=lYgU1nfuyjc), that imported fish captured in Brazilian Amazon water systems and bred F1 fish in Japan. In this species, both sexes develop individual-specific color patterns of many white lines or dots on their entire bodies (Fig 1A, 1C and 1D). Experimental fish were kept in aerated tanks (120 × 60 × 50 cm, 360 L) at a temperature of 26.5–27.5°C and a pH of 5.5–6.5, with a 12 L: 12 D light regime designed to reproduce their natural habitat. Eight fish in four pairs (Experiment 1) and 12 fish in six pairs (Experiment 2) were kept in two tanks for three months during which time no reproductive behaviors were observed. Fish were fed twice daily with mosquito larva and artificial food. Although body-morph, size and coloration do not differ between the sexes [24], the shapes of genital papillae are slightly different, and these differences were used for sex identification.

Male-female pairs were taken from the same stock tank and introduced into a ‘spawning tank’ (60 × 45 × 45 cm, 120 L), containing a block (5 × 10 × 10 cm) as a spawning site. When they spawned (50–300 eggs per clutch), they were regarded as forming a breeding pair. When spawning and breeding started, the contrast of the white line and body coloration became higher than before the start (Satoh pers. obs.).

### Description of social displays

Since the social behavior of this fish has not previously been described, we first observed their social interactions with pair-partners and non-partner fish in the spawning tank. Eight fish in four pairs (158.8 mm ± 4.1 (mean ± SD), *n* = 8) were used. In two pairs, after carefully removing one fish from the pair by hand-nets, we introduced a non-familiar fish (that focal fish had never seen) of the same sex and a similar size to the removed fish. We recorded the interactions between the original fish and the introduced fish using a video camera (HDR-CX390, Sony, also used in all video-recordings in this study) for 10 min. Next, we removed the non-familiar fish and reintroduced the partner, and 20 min after the reintroduction the interactions between the pair-partners was recorded for 10 min. In the other two pairs, we recorded the interactions in pairs for 10 min, and then recorded interactions between the partner fish and the replaced non-familiar fish for 10 min.

We observed four types of displays in this experiment: ‘head-down displays’ and ‘lateral displays’ occurred exclusively between pair-partners, while ‘frontal-displays’ and ‘mouth-bumping’ were observed only between non-familiar fish, regardless of sex (Table 1). Thus, the head-down display and lateral display (hereafter called partner-displays) indicate that fish recognize pair-partners, whereas frontal displays and mouth-bumping (hereafter non-familiar-fish displays) can be regarded as behaviors indicating that the performer regards the fish as a non-partner. In these displays fish often assumed a lateral position, regardless of whether the other fish was a partner or a non-partner.

**Table 1:** An ethogram of partner-displays (head-up display and lateral-display) and non-partner-displays (frontal display and mouth-bumping) by the discus fish *Symphysodon aequifasciatus*.

Partner-display	Head up display	Partner fish approach each other, holding up their heads and folding their pelvic fins to their body. When they swim away from one another, they lower their heads and spread their pelvic fins.
	Lateral display	A fish spreads all fins and displays the side of its body towards its partner.
Non-partner-display	Frontal display	A fish approaches the lateral side of the non-partner while opening its mouth and spreading its gill covers and fins.
	Mouth bumping	A fish aggressively bites and/or bumps the non-partner, often after frontal display.

### Experimental procedure

Photographs of each fish in lateral (both right and left sides) and frontal views were taken in the stock tank or the spawning tanks, when fish were stationary near the glass walls (Canon EOS Kiss X, Canon). An experimental tank (90 × 45 × 45 cm, 180 L) was prepared that had two compartments separated by an opaque board. The board had a small gate with a door through which fish could pass (Fig 1E). A 52 x 32 cm monitor display (MODEL-MOJ, DELL) was placed next to the left compartment. Digital photographs of fish were displayed on the monitor, so that focal fish in the compartment could see them. The size of the digital model displayed on the monitor was adjusted to the size of the partner. Fish responded to the displayed image as to real fish in many cases, and the fish social responses were analyzed to examine the prediction from the hypotheses. It is reported that cichlids have visual pigments sensitive from the ultraviolet to the red end of the spectrum [25]. We assumed the discus (Cichlidae) also has such sensitivity, but our camera and monitor did not cover ultraviolet.

#### Experiment 1

To test the ‘Encountering hypothesis’, of which prediction is largely different from those of hypotheses 2 and 3, we presented digital images of partners and non-partners in frontal and lateral views to focal fish (Fig 1B–1D). If the encountering hypothesis is applicable, fish should pay more attention to the frontal view of the fish and will use coloration of frontal head and face to distinguish familiar partners from non-partners.

For this experiment, we used the eight fish of the four pairs that were previously used for behavioral observation. One breeding pair was introduced into the experimental tank at a time (Fig 1E), and kept there for three days or more. During this time the monitor showed a film of an aerated aquarium without fish, so that the focal fish could become accustomed to the presence of the monitor. When only one member of the pair was in the experimental compartment (left), the door was closed, and experiment was conducted. Four types of models (frontal and lateral [left side] views of partner and non-partner) were displayed once in the center of the monitor for one minute at random order. We presented models at daily intervals for a period of one week, showing one model to each fish per day. The behavior of focal fish was video-recorded during the model presentation. After the presentation, the door between compartments was opened, and when each fish moved to the opposite compartment, we carried out the presentation for the other fish of the pair. Our preliminary experiments showed that both sexes had similar aggressive reactions to the image of the male non-partner model, and the model of the same male non-partner was displayed to both sexes in experiment 1. The experiment was carried out during daylight hours, from 12:00–15:00. Using the one-minute video recordings, we analyzed 1) the period of time during which the eye of the focal fish was within 5 cm of the model on the monitor, and 2) the number of four types of displays by the focal fish (Table 1). There were no differences between sexes in the focal fish for either the former metric (Mann Whitney U-test, *P* = 0.24 in all four cases) or the latter metric (*P* = 1.0 in all cases), so data from males and females were pooled.

#### Experiment 2

Twelve fish in six pairs (133.6 mm ± 4.2 SD, ranging from 125 to 141 mm, *n* = 12) were used in experiment 2. To test the ‘Main body’ hypothesis and ‘Face-specific’ hypothesis, we examined which coloration patterns serve as visual signals for individual recognition in *S*. *aequifasciatus*. If Main body hypothesis is correct, fish should use the color pattern on the trunk, as well as facial color. If Face-specific hypothesis is correct, fish will use only the coloration in the facial area. To this end, we prepared four types of models using Adobe Photoshop CS software according to the methods of Kohda et al. (2015): facial areas of the photographs were exchanged between fish photos (Fig 1B). Through this method, we created a “partner’s face (Pf) and partner’s body (Pb)” model (PfPb), “partner’s face and non-partner’s body (Nb)” model (PfNb), “non-partner’s face (Nf) and partner’s body” model (NfPb) and “non-partner’s face and non-partner’s body” model (NfNb). Non-partners were from photos of fish that focal fish had never seen and of the same sex to the partner. In the models where faces and bodies were exchanged, we carefully blended the region around the border of the face so that the graduation of color appeared to be natural. In preparing partner model (PfPb) and non-partner models (NfNb), we just cut and pasted their facial image in the PC for control. Face-specific hypothesis predicts that fish reactions should not differ between PfPb and PfNb or between NfPb and NfNb: that is, fish will use only facial coloration to distinguish among models. If fish do react differently to PfPb vs. PfNb or NfPb vs. NfNb, this would indicate that fish use only body coloration, not facial coloration as a visual cue, supporting Main body hypothesis. If the reaction to the modified models (PfNb and NfPb) will be intermediate between the reactions to partner (PfPb) and stranger (NfNb) models, this would suggest that fish use both facial coloration and other coloration patterns, also supporting the Main body hypothesis.

The procedure for the presentation of the models was identical to Experiment 1, except for the movement pattern of the model: the model appeared from the right edge of the screen and approached slowly, then swam to the left edge of the screen for a period of 30 seconds, then moved from left to right for 30 seconds, following the experiment design of Kohda et al. (2015). During the 60 second in which the model appeared on the screen, the behavior of the focal fish was recorded by video camera. Using these recordings, we analyzed the number and types of displays toward the digital models. There were no differences between the sexes of the focal fish in the frequency of partner- or non-partner-displays against the four types of models (Mann Whitney *U*-test, *P* > 0.14). We therefore pooled data from males and females. For statistical tests, non-parametric Wilcoxon signed rank test was calculated using R 3.0.1. Statistics Software (R Core Team, 2014). ANOVA was unavailable because of many zero values. Data used in these analyses are given in Supporting information files of this paper, and are available.

### Ethics statements

We did not sacrifice study animals during our experiments. We provided sufficient food and kept them in good aquarium conditions. Our experiments were conducted in compliance with the Animal Welfare Guidelines of the Japan Ethological Society, and were specially approved by the Animal Care and Use Committee of Osaka City University.

## Results

**Fig 2:**
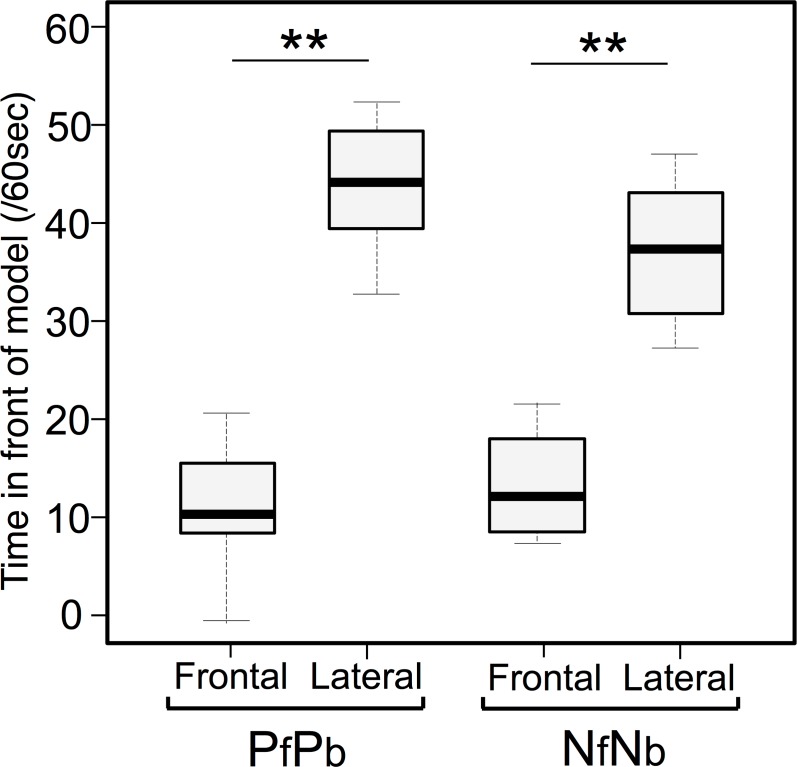
Time (sec) spent by focal fish in front of frontal and lateral views of partner (PfPb) and non-partner (NfNb) models presented on display monitors in Experiment 1. Median, box (showing 25% and 75%) and ranges are shown. ** *P* < 0.01 (Wilcoxon signed rank test).

In Experiment 1, focal fish spent much less time in front of the frontal-view models than the lateral-view models for both partner models (11.3 ± 2.1 seconds vs. 44.5 ± 2.2 seconds, respectively; Wilcoxon signed rank test, *T* = 0, *P* < 0.001, *n* = 8, Fig 2) and non-partner models (14.2 ± 2.3 second vs. 39.2 ± 2.5 seconds, *T* = 0, *P* < 0.007, *n* = 8). Focal fish spent less time in front of frontal-view models of partner (PfPb) and non-partner (NfNb) models, but the difference between the two types of models was not significant (*T* = 10.5, *P* = 0.29). Fish spent more time in front of lateral-view models, regardless of whether the model was of partner or non-partner models (*T* = 4.5, *P* = 0.11). Fish responded more frequently to lateral-view models than frontal-view models, regardless of whether the model was a partner or a non-partner.

**Fig 3:**
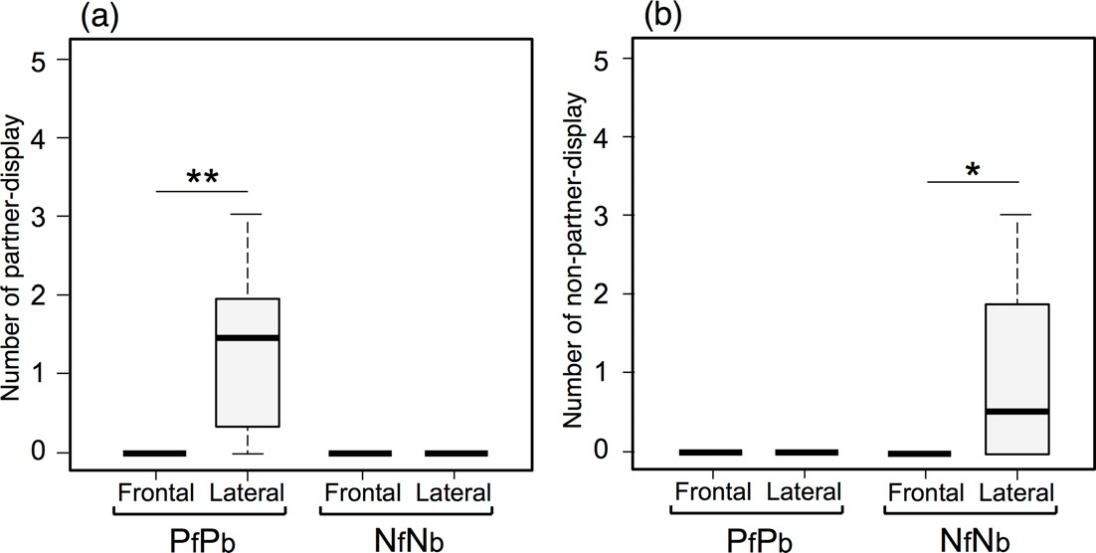
**Number of partner-displays to partner models (PfPb, a) and non-partner-displays against non-partner models (SfSb, b) in both frontal and lateral views in Experiment 1.** Partner-displays and non-partner-displays were not directed towards non-partner models and partner models, respectively. Median, box (showing 25% and 75%) and ranges are shown. ** *P* < 0.01, * P < 0.05 (Wilcoxon signed rank test).

In Experiment 1, fish conducted partner-displays frequently against lateral-view partner models, but not frontal-view partner models (Wilcoxon signed rank test, *T* = 0, *P* < 0.01, *n* = 8, Fig 3A). Focal fish did not conduct partner-displays against non-partner models. Fish frequently conducted non-partner-displays towards lateral-view non-partner models, but not frontal-view non-partner models (*T* = 0, *P* < 0.05, *n* = 8) or partner models (Fig 3B). These results suggest that fish are better able to distinguish between partners and non-partners using the lateral view. Since fish did not perform social displays toward any frontal-view models, we presented only lateral-view models to focal fish in Experiment 2.

**Fig 4:**
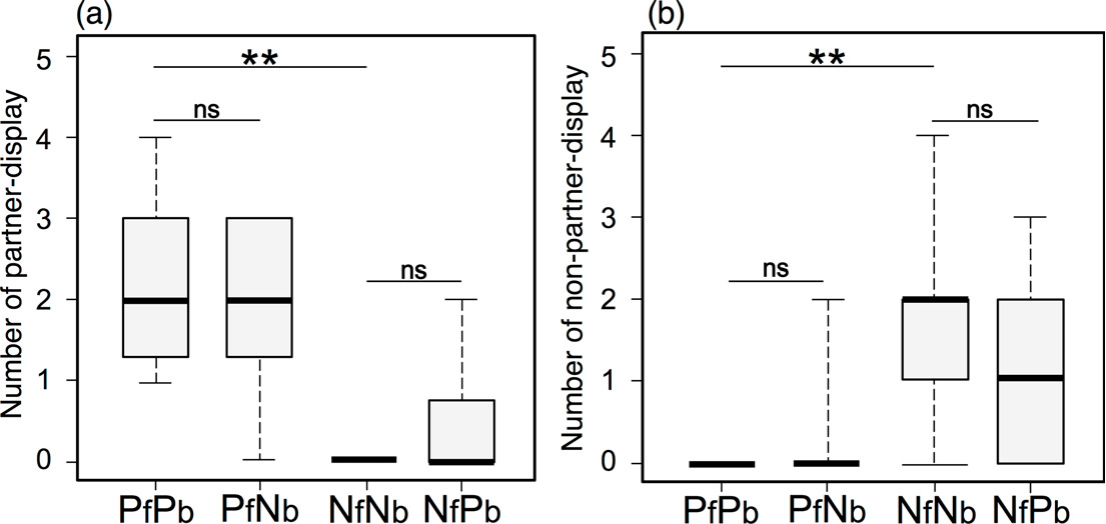
**Frequency of displays (min**^**-1**^**) against four types of models in Experiment 2: (a) partner-display and (b) non-partner-display.** Partner model (PfPb), partner face with non-partner body model (PfNb), non-partner model (NfNb) and non-partner face with partner body model (NfPb). Median, box (showing 25% and 75%) and ranges are shown. ** *P* < 0.01, ns *P* > 0.05 (Wilcoxon signed rank test).

Experiment 2 employed the partner (PfPb) and non-partner (NfNb) models, as well as two modified models (PfNb and NfPb) (Fig 4). As in Experiment 1, focal fish conducted frequent partner-displays toward partner models, but not non-partner models (Wilcoxon signed-rank test, *T* = 78.0, *P =* 0.002, *n* = 12, Fig 4A). Similarly, fish carried out non-partner-displays toward non-partner models but not to partner models (*T* = 0, *P* = 0.003, *n* = 12, Fig 4B), confirming that this fish are able to distinguish between partners and non-partners using the lateral-view images manipulated.

Partner-display behavior did not differ between models with partner face but different body (PfPb vs. PfNb, *T* = 41.0, *P* = 0.48, *n* = 12) or between models of non-partner face but different body (NfNb vs. NfPb, *T* = 0, *P* = 0.17, *n* = 12, Fig 4A). Furthermore, the frequency of non-partner-displays did not differ between models with the same face but different bodies (PfPb vs. PfNb, *T* = 0, *P* = 0.37; NfNb vs. SfNb, *T* = 34.5, *P* = 0.16, *n* = 12; Fig 4B). These results demonstrate focal fish only use facial coloration in discriminating between partners and non-partners, and are consistent with the Face-specific hypothesis. The frequency of partner-display and non-partner-displays toward modified models (PfNb & NfPb) was not intermediate between the frequencies of displays toward the original partner models (PfPb) and non-partner model (NfNb). These will not be consistent with the prediction from Main body hypothesis.

## Discussion

The discus fish *Symphysopdon aequifasciatus* has individual-specific color patterns on the entire body. The results of Experiment 1 showed that this fish is much more attentive to lateral-view models, and not performs social displays to frontal-view models of either partners or non-partners., Thus, this species does not ordinarily interact in a head-to-head position, being inconsistent with the Encountering hypothesis. Furthermore, since fish consistently conducted partner-displays toward partner models (PfPb) and non-partner-displays toward non-partner models (NfNb) laterally presented, this experiment showed that this fish is capable of distinguishing between pair partner and non-partner.

Experiment 2 provided evidence that more importantly they performed partner-displays toward partner-face models (PfPb and PfNb) with similar frequency, and also did non-partner-displays toward non-partner-face models (NfNb and NfPb) with similar frequency. These results clearly demonstrate that facial coloration is essential for fish recognition, and are consistent with the predictions of the Face-specific hypothesis. Fish performed non-partner-displays to NfPb and partner-display to PfNb models exclusively, indicating that they recognize them as non-partner and partner, respectively. Furthermore, the fact that responses to modified models were not intermediate between responses to PfPb and SfSb models indicates that fish do not use both facial and body coloration patterns for individual recognition. These results are consistent with the predictions of the Face-specific hypothesis, but not those from the Main body hypothesis. We can therefore conclude that facial coloration, but not other bodily traits, serves as a visual cue for individual recognition in *S*. *aequifasciatus*. This is the second documented instance of individual facial recognition in fish (see [15]).

When discus fish encountered frontal-view models, they would see only short lateral color lines on the head, and not see the entire ‘facial side’ (Fig 1C). This is likely to be the reason that the fish did not respond to frontal-view models. The cichlid *N*. *pulcher* and many other cooperative breeding cichlids and territorial damselfishes [14,15] have lateral facial coloration, but they lack frontal head coloration [15,26,27]. The balance of the evidence suggests that frontal head does not play a role for signaling individual recognition in these fish.

Conspicuous coloration often evolves in animals through sexual selection, where the sex that competes for mating opportunities will develop an attractive and showy morph [20,21]. The discus fish *S*. *aequifasciatus* is monogamous, and the coloration appears in both sexes during the breeding period after mating. It is therefore unlikely that the coloration patterns are a target of sexual selection. Facial coloration differs slightly among individuals, and serves as a social signal for individual identification between pair-partners. The facial coloration of the cooperative breeding cichlid *N*. *pulcher* appears in both sexes, and may be shaped mainly by natural selection [15,27]. Facial coloration in permanently territorial damselfishes appears regardless of sexes, and will be also used for individual recognition [14]. These examples suggest that social fish may often develop facial cues in both sexes to facilitate individual recognition, independent of mate-attraction.

Sexual selection, especially female choice, will induce sexual dichromatic coloration in males [20,21]. Sexually attractive color patterns in fish will often appear on the belly or trunk; e.g. three-spined stickleback (red belly), guppy (orange patch on trunk) and blue-headed wrasse (white patch on trunk) [28–30]. These sites are likely to be consistent with the Main body hypothesis. These signals contain information about physical conditions or genetic quality, and females will examine and compare the patch size and/or coloration or brightness among males [28–30]. Thus, the location of the signal colorations seems to be different depending on whether the signal is for mate attraction or individual recognition, but further comparative studies on the collation of the coloration of facial coloration and sexually attractive coloration are needed, and will induce interesting perspective on visual signal in social animals.

Eyes will play an important role in facial recognition in animals and human (e.g. fish [31,32] primates [33–35], human [36,37]). Research on eye movement shows that individuals tend to look at an opponent’s eyes before the rest of the face (e.g. humans [37], rhesus monkey [36], chimpanzees [7,37]). This suggests that if visual cues will be located near the eyes, such cues will allow rapid signal transmission. The Face-specific hypothesis for fish ([14,15] and present study) will be consistent with facial recognition patterns in mammals (e.g. [5–10]) and birds [10–12]. The cichlid *N*. *pulcher* distinguishes between familiar and unfamiliar faces within 0.5 seconds with high accuracy [15]). For this type of rapid and accurate facial recognition, individual-specific signals near the eye will be most effective. Despite the methodological difficulty of such studies, further research should examine eye movement in fish to further elucidate the mechanisms of facial recognition.

## Supporting Information

S1 File Data of the experiment 1. (PDF)

S2 File Data of the experiment 2. (PDF)
