# Supplementary material for: Facial Recognition in a Discus Fish (Cichlidae): Experimental Approach Using Digital Models
Source: PLoS One. 2016 May 18;11(5):e0154543. doi: 10.1371/journal.pone.0154543 (PMC4871422; doi:10.1371/journal.pone.0154543)
Supplement: S1 File — (PDF) [file pone.0154543.s001.pdf]

sample_no	turn	model	partner-display	non-partner-display	time front in model (sec)	sex
1	1	partner_frontal	0	0	10	m
1	3	partner_lateral	2	0	42	m
1	2	non-partner-frontal	0	0	22	m
1	4	non-partner-lateral	0	0	32	m
2	1	partner_frontal	0	0	12	f
2	2	partner_lateral	0	0	46	f
2	3	non-partner-frontal	0	0	21	f
2	4	non-partner-lateral	0	2	49	f
3	4	partner_frontal	0	0	21	m
3	1	partner_lateral	2	0	39	m
3	3	non-partner-frontal	0	0	10	m
3	2	non-partner-lateral	0	0	29	m
4	2	partner_frontal	0	0	11	m
4	1	partner_lateral	3	0	51	m
4	3	non-partner-frontal	0	0	9	m
4	4	non-partner-lateral	0	1	40	m
5	3	partner_frontal	0	0	9	f
5	2	partner_lateral	1	0	53	f
5	1	non-partner-frontal	0	0	13	f
5	4	non-partner-lateral	0	0	39	f
6	4	partner_frontal	0	0	17	f
6	3	partner_lateral	1	0	34	f
6	2	non-partner-frontal	0	0	13	f
6	1	non-partner-lateral	0	0	34	f
7	2	partner_frontal	0	0	11	m
7	1	partner_lateral	2	0	44	m
7	3	non-partner-frontal	0	0	15	m
7	4	non-partner-lateral	0	3	45	m
8	2	partner_frontal	0	0	0	f
8	4	partner_lateral	0	0	47	f
8	3	non-partner-frontal	0	0	11	f
8	1	non-partner-lateral	0	2	46	f
