# Supplementary material for: Facial Recognition in a Discus Fish (Cichlidae): Experimental Approach Using Digital Models
Source: PLoS One. 2016 May 18;11(5):e0154543. doi: 10.1371/journal.pone.0154543 (PMC4871422; doi:10.1371/journal.pone.0154543)
Supplement: S2 File — (PDF) [file pone.0154543.s002.pdf]

sample_no	turn	model	partner-display	non-partner-display	time front in model (sec)	sex
1	1	PfPb	4	0	42	m
1	3	PfNb	3	0	40	m
1	4	NfNb	0	1	32	m
1	2	NfPb	0	1	40	m
2	2	PfNb	2	0	42	f
2	1	PfNb	3	0	11	f
2	3	NfNb	0	2	32	f
2	4	NfPb	0	0	21	f
3	4	PfNb	1	0	32	f
3	3	PfNb	2	0	22	f
3	2	NfNb	0	3	44	f
3	1	NfPb	0	2	10	f
4	2	PfNb	2	0	10	m
4	4	PfNb	0	2	21	m
4	3	NfNb	0	1	27	m
4	1	NfPb	0	0	29	m
5	2	PfNb	3	0	46	m
5	3	PfNb	1	0	10	m
5	4	NfNb	0	4	11	m
5	1	NfPb	0	1	15	m
6	1	PfNb	3	0	32	f
6	2	PfNb	2	0	25	f
6	4	NfNb	0	2	25	f
6	3	NfPb	2	0	21	f
7	3	PfNb	3	0	27	m
7	1	PfNb	2	0	37	m
7	2	NfNb	0	2	39	m
7	4	NfPb	0	2	21	m
8	1	PfNb	2	0	10	f
8	3	PfNb	3	0	39	f
8	2	NfNb	0	2	22	f
8	4	NfPb	0	3	36	f
9	1	PfNb	1	0	35	f
9	3	PfNb	2	0	45	f
9	2	NfNb	0	1	39	f
9	4	NfPb	0	2	29	f
10	2	PfNb	1	0	38	m
10	3	PfNb	0	1	22	m
10	1	NfNb	0	0	9	m
10	4	NfPb	1	0	31	m
11	3	PfNb	2	0	44	m
11	2	PfNb	3	0	12	m
11	1	NfNb	0	2	36	m
11	4	NfPb	1	1	29	m
12	4	PfNb	2	0	49	f
12	1	PfNb	2	0	47	f
12	2	NfNb	0	1	44	f
12	3	NfPb	0	2	50	f
